# Obstacles to applications of nanostructured thermoelectric alloys

**DOI:** 10.3389/fchem.2014.00111

**Published:** 2014-12-18

**Authors:** Peter A. Sharma, Joshua D. Sugar

**Affiliations:** ^1^Quantum Phenomena, Sandia National LaboratoriesAlbuquerque, NM, USA; ^2^Materials Physics, Sandia National LaboratoriesLivermore, CA, USA

**Keywords:** microstructure, thermoelectrics, nanostructuring, precipitation, decomposition

A major theme in thermoelectric research is based on controlling the formation of nanostructures that occur naturally in bulk intermetallic alloys through various types of thermodynamic phase transformation processes (He et al., 2013). The question of how such nanostructures form and why they lead to a high thermoelectric figure of merit (zT) are scientifically interesting and worthy of attention. However, as we discuss in this opinion, any processing route based on thermodynamic phase transformations alone will be difficult to implement in thermoelectric applications where thermal stability and reliability are important. Attention should also be focused on overcoming these limitations through advanced post-processing techniques.

The primary effect of nanostructure formation is presently believed to be a reduction in the lattice thermal conductivity without a corresponding degradation in the electrical conductivity and Seebeck coefficient, resulting in large values of zT for a number of different alloys. It is also possible for nanostructures to increase power factor through an energy filtering effect (Faleev and Léonard, 2008). Due to the constraints imposed by effective medium theory (Bergman and Levy, 1991), the effective zT of a composite cannot be larger than any of its components under small temperature gradients. This means that any increase in zT of a material with small scale inhomogeneity must be attributed to transport across interfaces. A major problem with nanostructuring through phase transformations is that thermodynamic driving forces will always result in the elimination of interfaces and a corresponding reduction in zT over time at elevated temperatures. A related and less appreciated problem is that alloys with nanoscale microstructures are also likely to have inhomogeneities at much larger length scales. A system with different microstructural features at varying scales as opposed to uniform nanostructures has been suggested to have higher zT (Biswas et al., 2012). However, all the different microstructural features will evolve at elevated temperatures. In this circumstance, multiple microstructural features will interact in an unpredictable manner. There is no a priori reason why the driving force for coarsening will be reduced. Advanced post-processing steps can provide opportunities to overcome these limitations and potentially produce large volumes of material with a uniform, refined microstructure and higher reliability. The post-processing of inhomogeneous thermoelectrics is just as important as reaching the highest values of zT in materials with a particular type of microstructure.

Inhomogeneity at any length scale implies some sort of compositional or morphological variations that coexist within a bulk monolith. There are several ways of achieving inhomogeneity through thermodynamic driven processes, including: phase decomposition or precipitation, ordering, and diffusionless or martensitic transformations. An important question about these potential mechanisms for producing nanostructures is whether they result in a uniformly distributed nanostructure that maximizes the interface density over a large material volume. Any process that relies on nucleation and growth will face challenges in implementation because surface-energy driven effects lead to non-uniform nanostructures over large volumes of material with limited temperature stability. For example, if the initial nucleation of a new phase occurs heterogeneously at a particular defect, it is nearly impossible to redistribute that phase uniformly away from these defects. In addition, at elevated temperatures where diffusion is active, nanoscale precipitates will coarsen and cease to be nanoscale, reducing their effectiveness. The process where the average size of precipitates increases with time can occur through coalescence if the precipitates are mobile. If precipitates of varying sizes are present and diffusion is active, Ostwald ripening or coarsening will increase the size of the larger precipitates at the expense of the smaller ones. Processes that result from the growth of compositional instabilities such as spinodal decomposition are more promising in terms of long-range uniformity because there is no nucleation event, but still can suffer from coarsening and discontinuities at grain boundaries (Gronsky and Thomas, 1975). Martensitic transformations are promising in terms of uniformity and stability, but do not seem to occur in many compounds with a high thermoelectric figure of merit. A possible exception is Ag_2_Te (Manolikas, 1987).

The different possible strategies for using thermodynamics to nanostructure materials are shown in Figure 1. A typical, hypothetical binary phase diagram is shown in which M and N form a eutectic at approximately 50% N. First, consider solid-state precipitation of second phases as shown in Figures 1B,E. In both cases, the curvature of the solvus line allows a supersaturated solution of α or β to be fabricated. When temperature is reduced, second-phase precipitates form. In Figure 1B, the grain interiors show discrete and uniformly distributed small-scale precipitates. This type of microstructure with round, nanoscale precipitates uniformly distributed in the material is ideal for scattering phonons. However, at grain boundaries, the precipitates are larger in size as a result of heterogeneous nucleation, and the precipitates coarsen at elevated temperature, increasing in average size with longer anneal times. In Figure 1E, the precipitate morphology is different than that in Figure 1B as a result of elastic and surface energy contributions to the precipitate morphology. The precipitates are not round, but rather are flat plates, and their initial nucleation occurs on planar defects that accommodate compositional variations (similar to AgSbTe_2_-Sb_2_Te_3_; Sugar and Medlin, 2011). In this case, an interconnected second-phase distribution forms with an overall lower zT from an effective medium perspective that is also subject to coarsening with time at elevated temperatures. As a result of both heterogeneous nucleation and coarsening, these two examples show that solid-state precipitation can only have limited applicability for enhancing zT in bulk thermoelectrics. In very specific situations with the appropriate chemical, elastic, and interfacial properties, a system of uniformly distributed and distinctly separated round precipitates can form, but these types of nanostructures are still unstable at elevated temperatures for long times.

**Figure 1:**
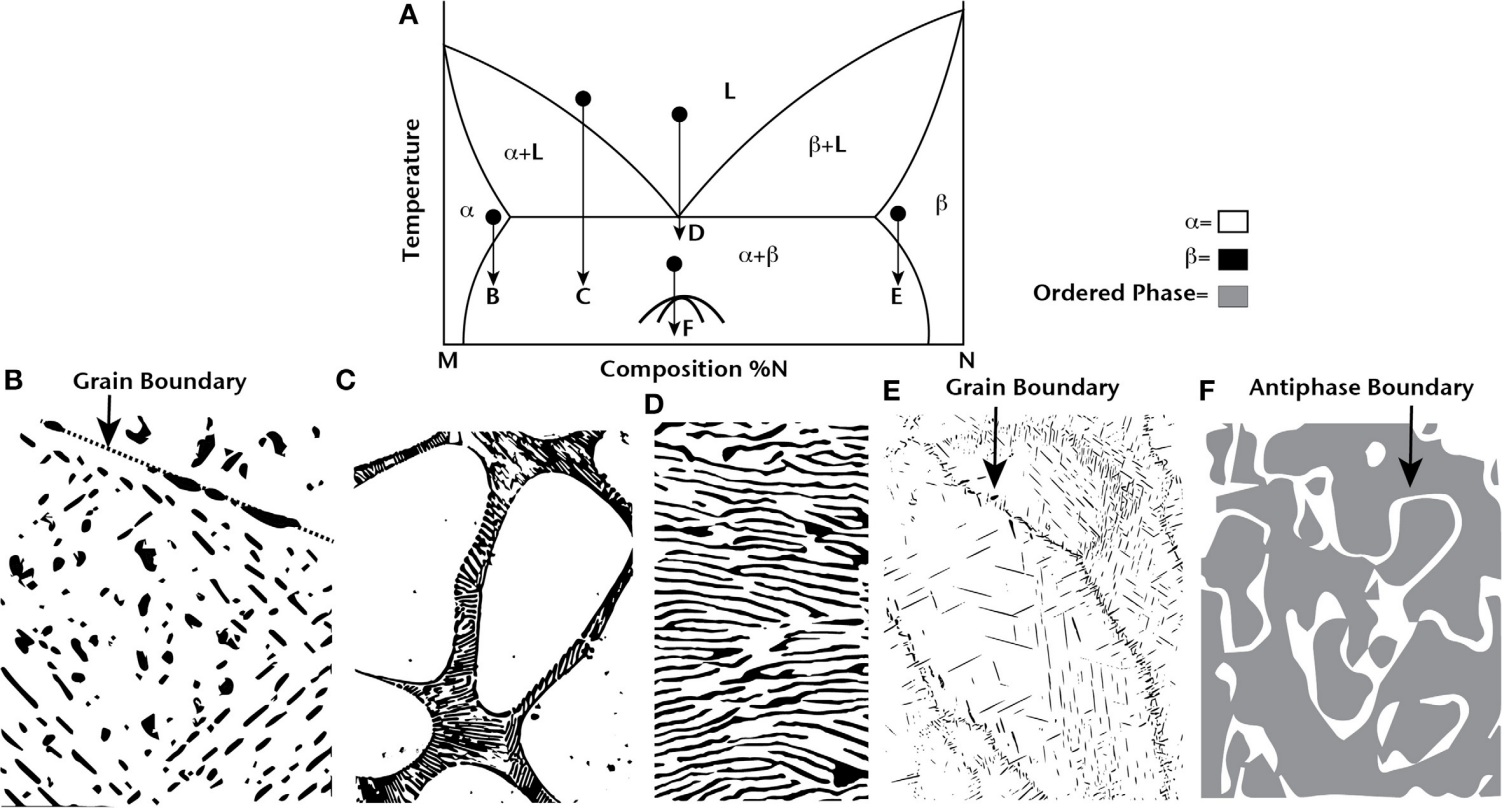
**A typical eutectic binary phase diagram for components M and N is shown in (A)**. Four different heat treatment strategies are schematically shown for producing nanostructured materials: solid-state precipitation **(B)** and **(E)**, hypoeutectic solidification **(C)**, eutectic solidification **(D)**, and ordering **(F)**. The challenges of these types of microstructures are discussed in the text.

Next, consider the hypoeutectic solidification (or hypereutectic depending on the labeling of the components) in Figure 1C. In this case, the solute rejection that occurs during solidification forces the nanostructured material to occur only at grain boundaries. As a result, the grain interiors are free of nanostructures. In addition, the nanostructured lamellae that occur at grain boundaries will still coarsen at elevated temperature. This microstructure also has limited use for enhancing thermoelectric properties.

Eutectic solidification seems more promising because the solidification process involves three-phase equilibrium, therefore it is easier to form a homogeneously distributed nanostructure over a large volume of material at appropriate solidification rates. However, at any temperature where diffusion is active, the lamellae will coarsen to remove interfaces and lower the total energy. Similarly, an ordering reaction like the one depicted in Figure 1F has the potential to be uniformly distributed throughout a large volume of material, but at elevated temperature there will be a driving force to reduce the number of antiphase boundaries and coarsen the domains.

Experimentally, each one of the situations depicted in Figure 1 have been explored. Examples include second-phase precipitates in PbTe (Lensch-Falk et al., 2010), AgSbTe_2_ (Burmeister and Stevenson, 1964; Sharma et al., 2010), and LAST (Perlt et al., 2013), eutectic solidification in PbTe (Ikeda et al., 2007), spinodal decomposition in PbTe-GeTe (Gorsse et al., 2011) and PbSnTe-PbS (Androulakis et al., 2007), and ordering in AgSbTe_2_ (Ma et al., 2013) and TAGS (Cook et al., 2007). All of these materials have high zT at elevated temperature and are being considered for power generation applications. Any of the phase transformations shown in Figure 1 can occur through two processes. First, the continuous diffusion of atoms down chemical potential gradients results in short-range ordering or segregation that eventually becomes long-range if the process is allowed to continue. This type of process is spinodal-like. Alternatively, the transformation process may occur through a discontinuous nucleation event, for which there is an energetic barrier, followed by the growth of the nucleated phase. Regardless of the mechanism, the processes that form nanostructures are prone to discontinuous reactions at defects, and these nanostructures remain stable only when diffusion is slow, at relatively low temperatures.

Given the challenges with controlling the nanostructure in multi-component thermoelectric compounds, it is unlikely that thermodynamic-driven processes implemented with simple heat treatments will be effective in producing reliable, nanostructured bulk materials. The value for zT is highly temperature dependent in any given material. If diffusion is active near the temperatures at which zT is large, precipitate coarsening will quickly eliminate any improvement in device efficiency. A strategy for fixing shape and size of precipitates over time at elevated temperatures is needed to make use of a high zT in this case.

An analogy can be found in precipitation hardened Al alloys, which are operated at low temperatures where diffusion is slow in order to retain their precipitate distribution and structure. At elevated temperatures, such alloys lose the mechanical properties for which they are designed. For thermoelectrics, such a strategy would only work for materials with an optimal zT at low temperatures, such as materials based on alloys of Bi or Bi_2_Te_3_. For higher temperature operation, approaches that pin interfacial motion with impurities or that produce special interfaces with decreased mobilities might be possible with more exotic processing techniques. To draw more analogies with metallurgical processing, structural metals are not normally used with an as-cast microstructure. In addition to heat treatments, forging, working, and hot/cold rolling of metals must be used to achieve a microstructure more suitable for structural applications. Similarly, other post-processing steps can be implemented in bulk thermoelectric materials to produce low mobility interfaces that can retain nanostructures at elevated temperatures.

Many studies of high zT materials often use advanced sintering techniques, such as field assisted sintering or hot pressing (Biswas et al., 2012), to achieve high density. Techniques such as equal channel angular extrusion (Im et al., 2004) and cryo-milling (Kuo et al., 2011) show promise as methods for refining the microstructure of bulk thermoelectric materials for homogeneity. These kinds of post-processing techniques are probably also useful for maintaining the stability of nanostructures. One way this might happen is that in the crushing and sintering processes, foreign species can be incorporated at grain boundaries that inhibit grain growth and can limit precipitate coarsening to dimensions commensurate with a single grain. In this scenario, the foreign species must have a small solubility in the material of interest at the operating temperatures of interest. Alternatively, post-processing might result in interfaces with particularly slow kinetics of motion even at elevated temperature, which would impede coarsening phenomena. A significant effort is needed to understand what types of interfaces show promise for having slow migration kinetics at typical operating temperatures. Although the synthesis of new bulk thermoelectrics with nanoscale inhomogeneity will probably result in the discovery of higher values of zT, these materials are not necessarily useful in their as prepared form. For long-term, reliable use of these materials, additional processing steps that go beyond simple heat treatments will be needed to retain and stabilize nanostructures over the desired range of operating conditions.

## Conflict of interest statement

The authors declare that the research was conducted in the absence of any commercial or financial relationships that could be construed as a potential conflict of interest.
